# Long-term genomic and epigenomic dysregulation as a consequence of prenatal alcohol exposure: a model for fetal alcohol spectrum disorders

**DOI:** 10.3389/fgene.2014.00161

**Published:** 2014-06-02

**Authors:** Morgan L. Kleiber, Eric J. Diehl, Benjamin I. Laufer, Katarzyna Mantha, Aniruddho Chokroborty-Hoque, Bonnie Alberry, Shiva M. Singh

**Affiliations:** Molecular Genetics Unit, Department of Biology, University of Western Ontario, London, ON, Canada

**Keywords:** epigenetics, neurodevelopment, mouse models, fetal alcohol spectrum disorders, DNA methylation, microRNA, histone modifications, gene expression

## Abstract

There is abundant evidence that prenatal alcohol exposure leads to a range of behavioral and cognitive impairments, categorized under the term fetal alcohol spectrum disorders (FASDs). These disorders are pervasive in Western cultures and represent the most common preventable source of neurodevelopmental disabilities. The genetic and epigenetic etiology of these phenotypes, including those factors that may maintain these phenotypes throughout the lifetime of an affected individual, has become a recent topic of investigation. This review integrates recent data that has progressed our understanding FASD as a continuum of molecular events, beginning with cellular stress response and ending with a long-term “footprint” of epigenetic dysregulation across the genome. It reports on data from multiple ethanol-treatment paradigms in mouse models that identify changes in gene expression that occur with respect to neurodevelopmental timing of exposure and ethanol dose. These studies have identified patterns of genomic alteration that are dependent on the biological processes occurring at the time of ethanol exposure. This review also adds to evidence that epigenetic processes such as DNA methylation, histone modifications, and non-coding RNA regulation may underlie long-term changes to gene expression patterns. These may be initiated by ethanol-induced alterations to DNA and histone methylation, particularly in imprinted regions of the genome, affecting transcription which is further fine-tuned by altered microRNA expression. These processes are likely complex, genome-wide, and interrelated. The proposed model suggests a potential for intervention, given that epigenetic changes are malleable and may be altered by postnatal environment. This review accentuates the value of mouse models in deciphering the molecular etiology of FASD, including those processes that may provide a target for the ammelioration of this common yet entirely preventable disorder.

## Introduction

The development of an organism from a single cell to a complex structure of distinct cell types that can interact, communicate, and respond to internal and external cues is an enigmatic process. What is known is that it is initiated by non-identical but complimentary maternal and paternal genetic contributions that comprise the diploid genome of the developing fetus, leading to the development of a complex organism comprised of differentiated organ systems. The development of the human brain is perhaps the most poorly-understood process, beginning early *in utero* and extending well into adolescence. Also, at each stage it is directed by precise spatial and temporal control of gene expression that may be influenced by external cues such as maternal gene products, micronutrient availability, and environmental molecules (Ellis et al., 2005). Decades of research have demonstrated that the fetal environment, particularly neurodevelopmental adversity, places individuals at higher risk for cognitive, behavioral, and social deficits (Shonkoff et al., 2009). At its extreme, an adverse developmental environment has been associated with later-life psychopathologies. Few disorders, however, have such a clear etiology as fetal alcohol spectrum disorders (FASD). This common yet entirely preventable set of cognitive and behavioral abnormalities are caused by alcohol's ability to pleotropically disrupt neurodevelopmental processes and resulting in altered brain function over the lifetime of an affected individual.

Despite overwhelming evidence that alcohol adversely affects the developing fetus, many women continue to drink during pregnancy. The reasons for this are varied: naïvité of potential consequences, increase in the prevalence of alcohol use among females of child-bearing age, addiction, or unawareness of pregnancy may all contribute to the growing number of children exhibiting FASD. Regardless, the pervasiveness of prenatal alcohol exposure (PAE) in North America is high, with an estimated rate of 1 in every 100 live births (Sampson et al., 1997; Chudley et al., 2005). This incurs staggering socio-economic costs (Stade et al., 2009), burdening healthcare, affected individuals, and their families. Indeed, the cognitive and behavioral impairments associated with FASD often lead to poor social and academic performance, higher rates of mental illness, and increased risk for delinquent behavior (Streissguth and O'Malley, 2000; Fast and Conry, 2004).

Expecting women of childbearing age to abstain completely from alcohol in western cultures is unrealistic. Yet, understanding the molecular mechanisms that underlie the origin, manifestation, and maintenance of FASD phenotypes may assist in the development of preventive and amelioration strategies to improve the outcome of affected individuals. The current review examines work conducted in our laboratory and others that seek to evaluate how ethanol exposure at different stages of neurodevelopment can trigger immediate changes in the brain leading to FASD. The current data have allowed us to propose an epigenetic model of neurodevelopmental disruption that may account for the causes and consequences of this prevalent disorder.

## Modeling FASD in mice

In order to understand how PAE may result in the epidemiological findings relating to FASD in human populations will require an understanding of how alcohol affects the molecular processes that guide neurodevelopment. This requires the development and use of effective animal models that can be reliably generated and that recapitulate the endophenotypes commonly observed in individuals with FASD. Much of our understanding of the genetic and epigenetic consequences of PAE has come from mouse models. These models generally fall into two categories. First, multiple groups have evaluated the effect of chronic voluntary maternal ethanol consumption throughout pregnancy by utilizing strains of mice with high ethanol preference or high vulnerability to the intoxicating effects of alcohol, such as C57BL6/J (B6) mice (Gilliam et al., 1989; Allan et al., 2003; Boehm et al., 2008; Kleiber et al., 2011). Pregnant B6 females continue to consume approximately 70% of their total liquid intake in the form of an ethanol solution, exposing the developing fetus to low-to-moderate levels of ethanol throughout gestation. The resulting offspring show subtle but consistent phenotypes relevant to FASD such as delays in the development of neuromuscular reflexes and coordination, increases in novelty-induced anxiety, and deficits in spatial learning (Kleiber et al., 2011). These models have face validity in that they likely represent a common pattern of alcohol consumption in humans that choose to drink while pregnant. However, they can make it difficult to ascertain the direct effects of ethanol on particular neurodevelopmental processes or at specific times. To address this, we and others have utilized a second type of exposure paradigm where a punctuated high dose (“binge”-like) treatment of ethanol is administered at a specific developmental time. These binge doses are administered typically during the mid-first, second, or third trimester human equivalent, with the first two given via injection or gavage to pregnant females and the latter given directly to neonate offspring due to the variation in human vs. mice neurodevelopmental timelines (Kleiber et al., 2013; Mantha et al., 2013). Such “binge” models have allowed us to evaluate how ethanol may disrupt the molecular processes active at each stage of brain development, and how these disruptions may translate to later-life phenotypic abnormalities.

## The initial effects of ethanol induce cellular stress leading to apoptosis or adaptation and cell survival

Multiple studies have documented the initial effects of ethanol exposure on immature brain cells. Perhaps most consistent is the finding that ethanol, particularly at high doses, is toxic to cells actively undergoing developmental processes such as neurulation, differentiation, migration, and synaptogenesis. A binge-like exposure to such cells can cause mass apoptosis in susceptible cell types in the developing brain, which has been observed in multiple brain regions and at multiple developmental stages (Ikonomidou et al., 2000; Light et al., 2002; Dikranian et al., 2005; Young et al., 2005; Zhou et al., 2011). It is interesting to note that susceptibility to ethanol-induced neurodegeneration varies with developmental stage. Certain regions show vulnerability early in brain development (first trimester equivalent), such as derivatives of the vetromedial forebrain and gastrulating mesodermal cells (Sulik, 2005) and others displaying sensitivity much later (third trimester), such as the hippocampus, cerebellum, corpus callosum, and regions of the prefrontal cortex (Olney et al., 2002). High ethanol doses early in gestation may ultimately result in congenital abnormalities, preterm births, or fetal death and spontaneous abortion (Harlap and Shiono, 1980). The effects of binge-drinking later in gestation may not display as cranio-facial abnormalities such as those associated with FAS, but as more specific neuroanatomical differences. Neuroimaging studies have consistently identified abnormalities in brain structure in individuals prenatally exposed to alcohol, such as reduced cerebral and cerebellar volume, hypoplasia of the corpus callosum, reduced hippocampal volume, and reduction of the caudate nucleus and basal ganglia (reviewed in Norman et al., 2009). Further, regional increases in cortical thickness and gray matter, as well as decreased volume and disorganization in white matter have been reported in individuals lacking the cranio-facial abnormalities required for an FAS diagnosis (Sowell et al., 2008a). Diffusion tensor imaging findings indicate that inter- and intraregional connectivity is also reduced in PAE individuals, significantly affecting the corpus callosum and tracts innervating the frontal, occipital, and parietal lobes of the cortex, as well as the hypothalamic-pituitary-adrenal (HPA) axis (Lebel et al., 2008; Sowell et al., 2008b; Fryer et al., 2009). Whether these abnormalities represent apoptosis of particular cell types and subsequent reorganization of cells or alterations in neurodevelopmental synaptic pruning is uncertain, but it is likely the result of some combination of the two.

Apoptosis is a normal developmental process that eliminates abnormally overactive and underactive neurons from the total cell population through distinct molecular pathways. However, alcohol can inappropriately trigger this process in the developing brain via its ability to act as an NMDA receptor antagonist and a GABAergic agonist. This process is associated with the activation of *caspase-3*, a hallmark of the intrinsic apoptotic pathway (Ikonomidou et al., 2000), that is dependent on Bax, a pro-apoptotic member of the Bcl-2 family, suggesting that mitochondrial release of apoptotic signals is critical to ethanol-induced neurodegeneration (Nowoslawski et al., 2005). Data from our laboratory suggests that this may be initiated by the general up-regulation of genes and pathways that drive apoptosis, including glutamate receptors (*Grin2a, Grin2b*), *Tgfb3, Foxo3*, and *Jun* (Kleiber et al., 2013). Gene ontology (GO) biological functions and pathways affected by ethanol (identified based on altered mRNA transcript profiling) are associated with the positive regulation of genes associated with apoptosis and cell membrane integrity, and down-regulation of genes associated with mitosis and biomolecule synthesis. These data corroborate that ethanol exposure initiates a strong stress response in developing cells that is designed to minimize energetically costly processes to maximize cell survival. It is clear that some developing neurons succumb to ethanol toxicity; however, certain cell types encounter terotagenic adversity and undergo molecular adaptation, and form the foundation of further development. These surviving cells may then undergo further mitosis, differentiation, and establish synaptic connectivity to eventually become part of the final functional network of the mature brain. Indeed, the molecular players that aid in these processes are expected, in part, to depend on the developmental timing of alcohol exposure.

## Alcohol and neurodevelopmental stress response: survival and adaptation

It is unclear why some cells succumb to apoptosis while others survive. But, those cells that survive represent a population that must reinitiate neurodevelopment and adjust their developmental trajectories to recoup, at least somewhat, the functionality of those cells that are lost. These cells must do so within a relatively limited amount of time, via alterations to gene expression patterns. We are only beginning to understand how this interruption to developmental cues leads to an altered pattern of gene expression and genomic regulation that results in a molecular “footprint” that, while established early during neurodevelopment, may be persistent throughout the lifetime of an alcohol-exposed individual.

Experimental evidence that ethanol effects include both short and long-term changes to gene expression is accumulating. Most of these studies, particularly within the last few years, have been concerned with the brain as a major target of ethanol and a driver of the long-term neurobehavioral and cognitive effects. Interestingly, the molecular changes that may occur following ethanol exposure seem to, in part, be dependent on the timing of the ethanol exposure.

### Early-gestation (first trimester) exposure: disruption of neurulation, structural remodeling, and epigenetic reprogramming

Most studies examining ethanol's effects on the human first trimester equivalent of brain development have focused on gestational days (GDs) 7–9 in mouse models as a representative model for early-gestational ethanol exposure. Ethanol-exposure during this stage of development may also lead to craniofacial abnormalities similar to human FAS (Sulik, 2005). Microarray studies, such as Hard et al. (2005) and Green et al. (2007), identified genes associated with ethanol exposure at GD 7 and 8, respectively. Hard et al. (2005) identified six annotated genes, all down regulated, involved in extracellular membrane remodeling, including *Timp4* and growth factor signaling gene *Bmp15*. Green et al. (2007) examined not only the gene expression changes that occur in the brain following ethanol exposure at GD 8, but also how genetic background may affect both physiological and genetic changes. C57BL6/J mice were found to be extremely susceptible to early-exposure craniofacial abnormalities as compared to C57BL6/N mice, but, interestingly, the infrequency of physiological abnormalities in the latter strain was not indicative of the strength of genetic response within the brain. These results may explain why individuals both with and without craniofacial abnormalities may be similarly cognitively affected by ethanol teratogenesis. Major pathways associated with early gestational exposure included down-regulation of ribosomal proteins and the up-regulation of glycolysis and the pentose phosphate pathway, alterations to cellular adhesion, integrity, and cytoskeletal pathways, including canonical Wnt signaling (Hard et al., 2005; Green et al., 2007). These findings are corroborated by a more recent study illustrating that ethanol exposure at GD 9 results in altered expression of genes associated with mRNA processing, protein synthesis ubiquitination, apoptosis (Downing et al., 2012). The results argue that early-gestation ethanol exposure disrupts cellular processes associated with cellular proliferation, survival, mitosis, and migration, which is consistent with the physiological phenotypes observed in these studies. Interestingly, early gestational exposure is also associated with alterations in a number of genes associated with epigenetic processes including methylation, chromatin organization, and remodeling, including *Ilf3*, a gene involved in chromatin remodeling, and *Hist3h2a* (Zhou et al., 2011; Downing et al., 2012). The disruption of these processes have long-lasting consequences on gene expression and, subsequently, neural function (Kleiber et al., 2013). Expression array analysis of adult (PD 60) mouse brain tissue following early-gestational ethanol exposure revealed the altered expression of genes involved in cellular assembly, proliferation, apoptosis, and tissue morphology. Many of these functions are associated with the altered expression of *Ntf3*, a canonical neuronal survival growth factor. Further, these long-term effects of trimester one-equivalent exposure include the decreased expression of genes that regulate endoplasmic stress response such as *Dnajjc3*, suggesting that the surviving population of cells may show reduced ability to navigate further environmental stressors and may be more vulnerable to future insults.

### Mid-gestation (second trimester) exposure: disruption of cellular migration and differentiation

At the end of the first trimester and throughout the second trimester, neural stem cells (NSC) produce a large proportion of what will become mature, adult neurons (Bystron et al., 2008). As such, the effects of ethanol exposure have the potential to be amplified by the high rate of cell proliferation and maturation that occurs during this period. Recent publications have examined the short and long-term effects of a binge-like exposure at GD 16 (Kleiber et al., 2013; Mantha et al., 2014), roughly equivalent to mid-gestation in humans in terms of active neurodevelopmental processes. Similar to early gestational exposure, the initial cellular response to ethanol at mid-to-late-gestation largely involves cellular stress response and apoptosis, and the altered expression of genes that regulate cell cycle. Interestingly, GD 16 exposure also triggers changes to genes involved in cell assembly and organization such as *Pip5k1b*, involved in actin polymerization, and cellular movement, such as *Ccl3* and *Ccnt1*. During trimester two, newly-generated cortical NSC migrate from the ventricular zone (VZ) to the cortical plate following mitosis (Noctor et al., 2004). Given that trimester two is a period of cellular reorganization and migration, it is logical that cellular signaling that guide these processes are particularly responsive to intra- and extracellular cues. Alterations to these genes may result in decreased proliferation rate of NSC, increased migration out of the VZ and into the subventricular zone (SVZ), and subsequently, a decreased thickness of this region (Miller and Nowakowski, 1991) that has been observed in humans with FASD. Interestingly, these NSC are not readily susceptible to ethanol-induced apoptosis (Prock and Miranda, 2007) but rather show increased migration and inappropriate differentiation patterns (Camarillo and Miranda, 2008). These reports are consistent with findings that suggest that genes associated with cellular differentiation, migration, and morphology remain altered at PD 60. We identified altered expression of *Dlx1* and *Dlx2*, among the earliest genes to be expressed in the SVZ and critical to interneuron differentiation and migration (Eisenstat et al., 1999; Ghanem et al., 2012).

### Late-gestation (third trimester) exposure: disruption of cellular communication and synaptic connectivity

The third trimester has been called the “brain growth spurt” due to the occurrence of a period of rapid synaptogenesis during which much of the basis of cell-to-cell communication that will form adult neural circuitry is established. This period is also extremely sensitive to the ability of ethanol to trigger neurodegeneration, with a large proportion of cells observed to undergo apoptosis in numerous regions such as the cortex, cerebellum, corpus callosum (Olney et al., 2000). This is, in part, attributed to ethanol's ability to disrupt glutamatergic and GABAergic signaling. In rodents, synaptogenesis occurs during the first 2 weeks of neonatal life, with the peak occurring at approximately PD 7 (Dobbing and Sands, 1979). Given this difference in neurodevelopmental timelines between mice and humans, ethanol exposure during the third trimester can be modeled by early neonatal ethanol treatment in mice. Similar to first trimester exposure, the initial response to ethanol at this developmental stage is characterized by cellular stress, including an up-regulation of genes associated with apoptosis and a down-regulation of genes involved in energetically costly processes such as protein synthesis and mitotic progression. This is also associated with reduced expression of a number of growth factors such as *E2f4, Egr3, Egr4*, and *Vegfa*. Aside from cellular stress, ethanol affects the expression of a number of genes relevant to synaptic formation and maintenance, including *Cpeb1, Gabra5, Grin2a*, and *Grin2b*. Given that the formation of functional neural circuits is dependent on the synchronous activity of glutamate and GABA signaling, alterations to these genes likely disrupt the establishment of normal synaptic connectivity (Kleiber et al., 2013).

Additionally, given that much of the brain has undergone substantial differentiation by this stage, it is likely that ethanol affects gene expression in a particularly region-specific and cell type-specific manner. In particular, the hippocampus and the HPA axis appears to be susceptible to third trimester exposure as evidenced by the impairments in cognitive and behavioral phenotypes consistently associated with late-gestation (in humans) and early neonatal (in mice) ethanol exposure. Studies have identified alterations in NMDA and GABA subunit receptor expression and function immediately following neonatal ethanol exposure as well as into adulthood (Mameli et al., 2005; Toso et al., 2006; Puglia and Valenzuela, 2010; Kleiber et al., 2013). This is associated with impairments in the formation of organized synaptic connections and persistent deficits in long-term potentiation, explaining the consistent observation of impaired learning and memory formation in mouse models of FASD as well as affected individuals.

Other consistently-identified gene pathways altered shortly after ethanol exposure that remain altered into adulthood include endocannabinoid and retinoic acid signaling (Kleiber et al., 2013; Subbanna et al., 2013a). Retinoic acid receptor signaling has also been implicated in ethanol's effects on HPA axis formation and reactivity. Specifically, ethanol has been shown to affect steroid hormone signaling, including the immediate and long term dysregulation of thyroid hormone/retinoid X receptor signaling, propiomelanocortin, and Period gene expression (Chen et al., 2006; Kleiber et al., 2013). Interestingly, this effect is most pronounced in animal models exposed during the brain growth spurt period (Earnest et al., 2001; Sakata-Haga et al., 2006). Phenotypically, this results in altered Circadian rhythm and gluccocorticoid signaling that is associated with increased stress reactivity and vulnerability to anxiety, depression, hyperactivity, and diminished cognitive function, all of which are consistently observed in individuals exposed to ethanol during neurodevelopment (Earnest et al., 2001; Girotti et al., 2007; Weinstock, 2010).

### Continuous moderate exposure throughout gestation: evidence for no safe amount?

In our research we have not only modeled high-dose fetal alcohol exposures at specific neurodevelopmental times (via maternal or neonate injection) but also low-to-moderate chronic exposure by means of voluntary maternal consumption. Results from these studies are critical in our understanding of how alcohol affects the brain in ways that may not be obvious at an epidemiological or clinical level. There is evidence that specific neurodevelopmental times are particularly sensitive to ethanol teratogenesis and that significant neuroapoptosis can be triggered by a transient small increase in blood alcohol concentration (Young and Olney, 2006), leading to subtle but significant phenotypic consequences. Our group has contributed to these data by generating a mouse model of continuous gestational moderate alcohol exposure (Kleiber et al., 2011). The adult offspring exposed to ethanol using this paradigm exhibit subtle but consistent alterations to not only behavior, but transcriptomics and epigenetic patterning (Kleiber et al., 2011, 2012; Laufer et al., 2013). Such results imply that even low-to-moderate alcohol exposure can negatively affect neurodevelopment, leading to increased risk for behavioral and cognitive alterations. Interestingly, the effect of any prenatal alcohol exposure may be detectable by subtle but consistent transcriptomic and epigenetic changes. The results from these low-dose studies argue that neurodevelopment is highly susceptible to ethanol and that ethanol exposure, even at low doses, may produce long-term effects. Further, reports in mice have also suggested a transgenerational inheritance of fetal alcohol effects in subsequent unexposed generations (Govorko et al., 2012). These findings, if established in human populations, will impose yet another layer of complexity in FASD as a public health issue.

## Epigenetic mechanisms underlying molecular adaptation to neurodevelopmental ethanol exposure

Individuals born with FASD show phenotypes that persist throughout their lifetime. However, not all fetuses exposed to alcohol develop clinical manifestations of the disorder. We hypothesize that this variability may be related to the threshold of neurotranscriptomic changes that induce deficits in neurulation, cellular migration and differentiation, and synaptic development. Also, this may determine phenotypic severity, which ranges from fetal death to subtle or no obvious effects. Indeed, it has been suggested that the variety of molecular and cellular responses to neurodevelopmental ethanol exposure is likely to involve a “potentially bewildering array of heterogeneity” (Haycock, 2009). Further adding to this variability is the known effects of alcohol on epigenetic mechanisms (Shukla et al., 2008).

Studies have established that a fundamental change in the adult transcriptome persists beyond the cessation of alcohol exposure and developmental processes (Chang et al., 2012; Kleiber et al., 2012, 2013), and attention has been turned to the processes that may regulate and maintain these changes. Specifically, the impact of prenatal alcohol exposure on developmental epigenetic processes have generated a number of important recent reviews, dealing with the topic from molecular and clinical perspectives (Haycock, 2009; Resendiz et al., 2013; Ungerer et al., 2013). Alcohol-induced alterations to epigenetic processes may strongly impact normal developmental and adult brain gene expression. These processes can have transient or long-lasting effects, meaning that ethanol-induced disruption of the establishment of epigenetic patterning will also be long-lasting. On-going studies have implicated both DNA methylation, histone modifications, and non-coding regulatory RNAs (ncRNAs) in the effects of neurodevelopmental ethanol exposure.

### DNA methylation and genomic imprinting

Development includes dynamic epigenetic changes, including genome-wide demethylation following oocyte fertilization prior to implantation and *de novo* genome methylation during gastrulation that continues to be established in a cell-specific, tissue-specific, or parent-of-origin manner (Reik et al., 2001). The alteration of DNA methylation patterning, occurring at CpG dinucleotides and within CpG islands to control gene activation, provides a potential target for ethanol to alter gene expression via epigenetic regulatory control, including within developmentally imprinted regions. Ethanol interferes with one-carbon metabolism, the primary methyl donor in the DNA-methyltransferase pathway, and it has been shown that one-carbon metabolism is indeed impaired by ethanol exposure in rodent models (Halsted et al., 2002; Fowler et al., 2012). This is accomplished, in part, by reducing folate availability. Folate is converted in a step-wise process to methionine, which is then converted to the active methyl donor S-adenosylmethionine (SAM). Reductions in SAM impair the ability of DNA methyltransferases (DNMTs) to maintain DNA methylation. Ethanol can also reduce SAM levels by reducing the activity of methionine synthase (Barak et al., 1996).

As early as 1991, Garro et al. (1991) demonstrated that gestational ethanol exposure resulted in genomic hypomethylation and reduced methylase activity. More recently, DNA methylation studies have shown that adult mice prenatally exposed to ethanol show alterations in known methylation-sensitive genes (Kaminen-Ahola et al., 2010) and show broad alterations when examined at the whole-genome scale, including within imprinted regions (Laufer et al., 2013). These results concur with other studies reporting DNA methylation changes in genes that are known to be genomically imprinted following prenatal alcohol exposure (Liu et al., 2009). Imprinted genes are expressed in a parent of origin-specific manner that is based on differential methylation of an imprinting control region (ICR). Imprinting is critical during neurodevelopment, as well as in the normal functioning of the brain (Davies et al., 2008). Further, approximately 30% of parentally-imprinted transcripts are hypothesized to be non-coding RNAs (ncRNA), meaning that ethanol-induced methylation changes can cause long-term changes in gene regulation at both the level of transcription and translation (Morison et al., 2005). Interestingly, many sequences vulnerable to ethanol-induced methylation changes possess CCCTC-binding factor (CTCF) sites, a transcription factor that is sensitive to the methylation status of its binding sequence. CTCF motifs control the parent-of-origin-based expression of many ICRs through the binding of CTCF, an insulator zinc-finger protein. Previous research has found that the CTCF binding sites in *H19/Igf2* ICRs show significantly altered methylation patterns in ethanol-exposed placental tissue (Haycock and Ramsay, 2009). Also, subtle changes to *Igf2* locus DNA methylation and expression following prenatal alcohol exposure have also been reported (Downing et al., 2011).

The *H19/Igf2* region was also identified by Laufer et al. (2013), which reported altered methylation in the adult brain of mice prenatally exposed to moderate chronic alcohol throughout gestation. In this study, analysis of the upstream sequences of 30 genes with altered expression within the adult brain of prenatally-exposed mice indicated that 12 (40%) showed sequences that were strongly predicted to be CTCF binding motifs. Among these were genes associated with canonical PTEN/AKT/mTOR signaling, with 57% of molecules involved in the *Pten* pathway showing significant differential methylation and a gain of methylation observed at a predicted CTCF-binding site within the promoter region of *Akt* (Laufer et al., 2013). This pathway regulates a number of neurodevelopmental processes such as morphogenesis, dendritic development, synapse formation, and synaptic plascticity (Yoshimura et al., 2006). This results are interesting in light of previous gene expression and protein activity studies that have suggested PTEN/AKT signaling as a potential initiation point for the actions of ethanol on the developing brain (Xu et al., 2003; Green et al., 2007). These data suggest that this site, and consequently imprinted regions of the genome that utilize CTCF as an insulator, may be particularly vulnerable to methylation alterations following neurodevelopmental alcohol exposure. This would argue that ethanol has the ability to alter the expression of numerous genes via altered methylation patterning as well as via altered control of small ncRNAs present within imprinted genomic regions. These epigenetic changes may underlie the longevity of the gene expression changes observed by transcriptomic studies. Ultimately, these data suggest that changes in DNA methylation, particularly within imprinted regions that play critical roles in neurodevelopment and brain function, may have a role in the long-term maintenance of altered gene expression and cognitive endophenotypes associated with FASD.

### Histone modifications

Studies evaluating the involvement of histone modifications in prenatal alcohol exposure phenotypes are rather preliminary, though the relevance of histone modifications as a molecular consequence of alcohol abuse has been established (Kim and Shukla, 2006; Pal-Bhadra et al., 2007; Shukla et al., 2008). Ethanol exposure during the human third trimester-equivalent has been shown to alter histone acetylation in the developing rat cerebellum (Guo et al., 2011). Also, ethanol-induced hippocampal neurodegeneration induced on PD 7 in mice is achieved in part by the enhanced activity of G9a (lysine dimethyltransferase) and increased levels of histone H3 lysine 9 (H3K9me2) and 27 (H3K27) dimethylation (Subbanna et al., 2013b). Work in NSC has also found that ethanol exposure leads to reductions in H3K27me3 and H3K4me3 at promoters of genes involved in neural precursor cell identity and differentiation (Veazey et al., 2013). Many of these genes also showed corresponding changes in gene expression. Further, HDAC mRNA levels (Kirpich et al., 2012), protein levels (Kirpich et al., 2013), and protein function (Choudhury and Shukla, 2008) have been shown to be affected by ethanol exposure, including within our own studies (Kleiber et al., 2013). Importantly, some results have relevance to FASD-specific behavioral phenotypes. For instance, a recent report by Bekdash et al. (2013) showed that prenatal ethanol exposure resulted in decreased histone activation marks (H3K4me3, Set7/9, acetylated H3K9, phosphorylated H3S10) and increased repressive marks (H3K9me2, G9a, Setdb1) associated with hypothalamic *pro-opiomelanocortin* (*Pomc*) regulation, resulting in decreased *Pomc* expression and a heightened cortisol response. These results suggest an association between prenatal alcohol exposure, histone modifications, and HPA-associated phenotypes relevant to FASD.

### MicroRNAs

There is substantial evidence that microRNAs (miRNAs) are heavily involved in mammalian neurodevelopmental processes including cell proliferation, apoptosis, differentiation, synapse formation, and remodeling (Coolen et al., 2013; Nowak and Michlewski, 2013; Hu et al., 2014). In 2007, Sathyan et al. (2007) first explored of the role of regulatory miRNAs in the teratogenic effects of ethanol on the developing brain. This study reported the potential interplay of miR-9, miR-21, miR-153, and miR-335 miRNAs and their mRNA, illustrating the delicate yet sensitive balance between antagonistic biological cues that may ultimately determine cellular apoptosis or survival and adaptation following ethanol insult. Importantly, this study identified that miRNAs serve as an effective intermediary between a teratogen and cellular response as they are able to affect the regulation of numerous genes and developmental pathways in a complex and divergent manner.

We have employed a genome-wide strategy of interrogating miRNA: mRNA transcript relationships. Our results show that ethanol exposure during both trimester two and three-eqivalents results in the alteration of expression of a number of miRNAs (Table 1) (Mantha et al., 2014). A number of developmental processes, including cell maturation, are guided by miRNA-based control of transcript regulation. Of note is miR-335, found to be down-regulated in the adult brain following late-gestation ethanol exposure, and shown to be ethanol-sensitive in NSCs and regulates NSC differentiation (Sathyan et al., 2007; Mantha et al., 2014). Further, we also identified miR-10b to be down-regulated in the adult brain, which has been previously identified as ethanol-responsive (Wang et al., 2009). This miRNA is a regulator of the Hox gene family, which plays a key role in neuronal migration (Geisen et al., 2008). Similarly, the miR-302 family of miRNAs involved in cell cycle progression and the maintenance of embryonic stem-cell pluripotency, potentially through its interactions with WNT signaling (Groenendyk and Michalak, 2014).

**Table 1:** **miRNAs and predicted mRNA targets with inversely correlated alterations following neurodevelopmental ethanol exposure**.

**Treatment days**	**miRNA**	**Expression change^#^**	**Predicted mRNA target(s)**	**Expression change^#^**
GD 8/11^†^	miR-1192	↑	Atf1, Gng4, Map3k1, Rpe, Setd2, Stxbp6, Zc3h6	↓
	miR-532-5p	↑	Atf1, Itpripl2, Stxbp6	↓
GD 14/16^*^	miR-10b	↓	Aak1	↑
	miR-184	↓	Myl9	↑
	miR-302c	↑	Ccdc6, Mfap3, Ptpro, Rnd3, Rpl36a/r, Sema3c, Stoml3, Supt3h	↓
	miR-342-5p	↓	Aak1, Cables2, Rhog	↑
	miR-343	↑	Asic4, Dcn, Gpr116, Ptpro, Stoml3	↓
	miR-449b	↓	Ina	↑
PD 4/7^†^	miR-26b	↑	Adam9, Chsy1, Cnr1, Exoc8, Hs6st1, Lingo1, Map3k7, Mras, Pfkfb3, Ppm1b, Rhou, Sema6d, Shank2, Tab3, Tdrd7, Ube2j1	↓
	miR-34b-5p	↓	Kitl	↑
	miR-184	↑	Ncor2, Prkcb	↓
	miR-721	↑	Akap11, B4galt, Cnr1, Efnb2, Fam20b, Ino80, Irf1, Lrrk2, Ncoa3, Pfkfb3, Ppargc1a, Rbm9, Shank2, Spen, Sphk2, Tsc1, Wdfy3	↓
	miR-1970	↓	Arhgap6	↑

# Significance for expression change was 1.2-fold, p < 0.05.

* detailed data published in Mantha et al. (2013).

† data unpublished.

Analysis of alcohol-induced miRNA expression changes following third trimester-equivalent exposure yielded a slightly larger list of altered miRNAs (Table 1). Five of these showed an inverse relationship to a number of putative gene targets, involved in a number of neurodevelopmental process including corticotrophin and retinoic acid signaling, both critical to HPA axis development and function, as well as PI3/AKT/mTOR signaling (Kleiber et al., 2013; Laufer et al., 2013). Specifically, PI3K/AKT/mTOR signaling may be altered by the up-regulation of miR-721 and the down-regulation of its target, the tumor suppressor protein *Tsc1*, which has been associated with impairments in the migration and developmental positioning of pyramidal neurons in the hippocampus leading to cognitive function, learning, and memory deficits (Orlova and Crino, 2010; Mejia et al., 2013). Other studies have implicated specific miRNAs depending on cell type or ethanol treatment paradigm and our results have replicated some of these same molecules, including miR-335 [identified by Sathyan et al. (2007)] and miR-10b [identified by Mantha et al. (2014) and Wang et al. (2009)]. These results provide insight into how ethanol may alter the expression of numerous genes through the altered regulation of a select group of miRNAs. Further, a given biological pathway or process may be affected from multiple vantages simultaneously via the alteration of a few miRNA species, acting in an antagonistic and/or synergistic manner. We are truly only beginning to understand the regulatory control of miRNAs within the brain, but these results support the role of miRNAs in the neurodevelopmental alterations that follow prenatal alcohol exposure.

### The interplay of epigenetic factors in FASD-associated genomic dysregulation

**Figure 1:**
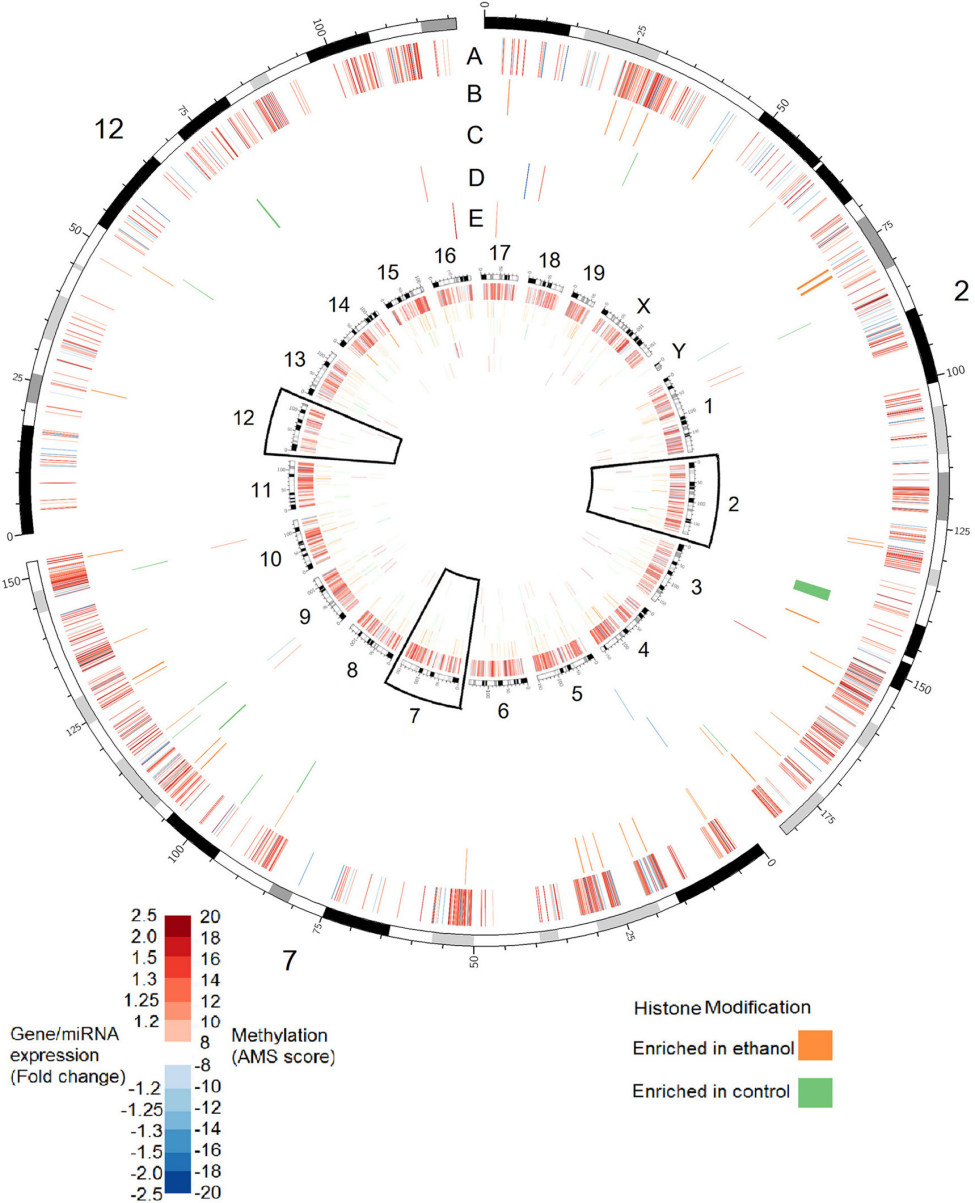
**Alterations in DNA methylation, histone modifications, miRNA expression, and gene expression in the hippocampus of adult (PD 60) mice exposed to ethanol during the trimester three equivalent (PD 7)**. Tracks show alterations in: (A) DNA methylation as measured by absolute methylation score (AMS); (B) Histone H3 lysine 27 trimethylation; (C) Histone H3 lysine 4 trimethylation; (D) miRNA expression; (E) Gene expression. Inner circle shows changes for all chromosomes. Outer circle shows an expanded view of chromosomes 2, 7, and 12, which contain major imprinting centers. DNA methylation significance was determined by the MEDME algorithm using an AMS *p*-value cutoff *p* < 0.05; miRNA and mRNA cutoff: *p* < 0.05, fold-change > 1.2.

Although these data point to a substantial role of epigenetic modifications in FASD etiology, research has not attempted to understand these changes in the larger context of the complete epigenetic landscape. Such an approach is important since epigenetic modifications do not operate in isolation; often, modification cross-talk is vital for function. Further, DNA methylation, histone modification, and ncRNA can co-regulate each other in complex regulatory networks (Sato et al., 2011). In order to address this, we characterized changes in four epigenetic processes and gene expression in the hippocampus of mice exposed to alcohol during the third trimester-equivalent. This analysis was performed in three stages: examination of DNA methylation at known promoters using methylation DNA immunoprecipitation followed by hybridization to genome arrays (MeDIP-chip); histone methylation analysis at H3K4me3 and H3K27me3 sites, which are, respectively, positively, and negatively correlated with gene expression (Barski et al., 2007); and miRNA and mRNA transcript profiling using expression arrays. Since the hippocampus is important for learning and memory, and its structure is affected by neonatal ethanol exposure, it is possible that ethanol may induce molecular changes in the hippocampus that are relevant to the learning and memory deficits observed in animal models and humans affected with FASD. Our MeDIP-chip results identified over 10,000 regions were differentially methylated (MEDME AMS algorithm, *p* < 0.05), with approximately 100–200 regions differentially enriched for H3K4me3 and H3K27me3. The results shown in Figure 1 show the genomic position of these changes in association to 40 miRNA and 60 mRNA transcripts shown to be differentially expressed following ethanol exposure in the hippocampus. These data, while admittedly preliminary and needing further examination, suggest that widespread epigenetic changes occur across the genome following neurodevelopmental ethanol exposure, and that the molecular factors that underlie the changes to neural gene expression and function are multifaceted and complex. They suggest that epigenomic dysregulation represents an integral aspect of prenatal alcohol exposure response that contributes to the development of FASD.

## The role of postnatal environment in phenotypic outcomes associated with FASD

Children with PAE are often raised in suboptimal conditions, but the effect of this stress has not been adequately explored. Mammalian offspring are fully dependent on maternal care during the early postnatal period. In this way, the quality and quantity of maternal interaction or caregiving poses a strong environmental influence of stress-related gene expression (Korosi and Baram, 2009). Protective factors against FASD in humans include a stable home environment, infrequent changes in living arrangement and non-exposure to violence (Streissguth et al., 1994). Maternal separation models are often used to model chronic early life stress, whereby 3 h of separation per day from postnatal days 2–14 can result in anxiety-like behaviors in adult mice that affect epigenetic patterning (Franklin et al., 2010). Following maternal separation as a stressor, mice display increased anxiety-like behaviors on open-field testing (Romeo et al., 2003) similar to those observed in PAE models. We have determined that prenatal alcohol exposure, particularly during the third trimester equivalent, alters a number of genes and pathways associated with HPA axis development, and that these pathways are among the few that remain altered significantly into adulthood (Kleiber et al., 2013). These include altered regulation of *pro-piomelanocortin* (*Pomc*), *Nr4a1*, and genes associated with thyroid hormone/retinoid X receptor function. Alterations to these pathways have been associated with HPA axis reactivity and are associated with increased risk for depression, anxiety, and poor coping skills related to exposure to later-life stressors, which have been consistently associated with prenatal alcohol exposure (Hellemans et al., 2008; Weinberg et al., 2008). Indeed, these studies have shown that PAE leads to later-life vulnerability to stress that is associated with changes to HPA axis function, with both hormonal and behavioral consequences.

The outcome of prenatal ethanol exposure appears to depend significantly on neonatal environment. A high-stress, unpredictable environment may increase the severity of manifestation of FASD-related phenotypes, while a stable, enriched environment may ameliorate them. Rehabilitative therapies in children with FASD currently aim to develop verbal, math and social skills concurrent with counseling sessions and specialized classes (Peadon et al., 2009; Kodituwakku, 2010). These effects may also be assessed in mouse models of FASD and typically include exposure to to physically and cognitively challenging or “enriched” postnatal environments. Rodents exposed to alcohol during neurodevelopment but postnatally reared in enriched environments show less susceptibility to novelty-induced stress and improved memory performance (Hannigan et al., 2007). Given how fetal alcohol exposure affects neurodevelopment, it is possible that the functional effectiveness of these enriched environments result from a targeted activation of specific molecular mechanisms that modify neural structure and function and are ultimately expressed as “rehabilitated” behaviors. Our lab has assessed the behavioral recovery of mice exposed to alcohol during neurodevelopment that are raised postnatally in either an enriched or neutral (standard) environment. The results suggest that at least some aspects of these FASD-specific alterations may be ammeliorated by engaging affected pups cognitively within an enriched environment, including decreased anxiety-related traits in the elevated plus maze assay and improved memory of novel and familiar objects. It will be valuable to assess if these phenotypic corrections have genetic and epigenetic correlates.

## Developing a working model for FASD

**Figure 2:**
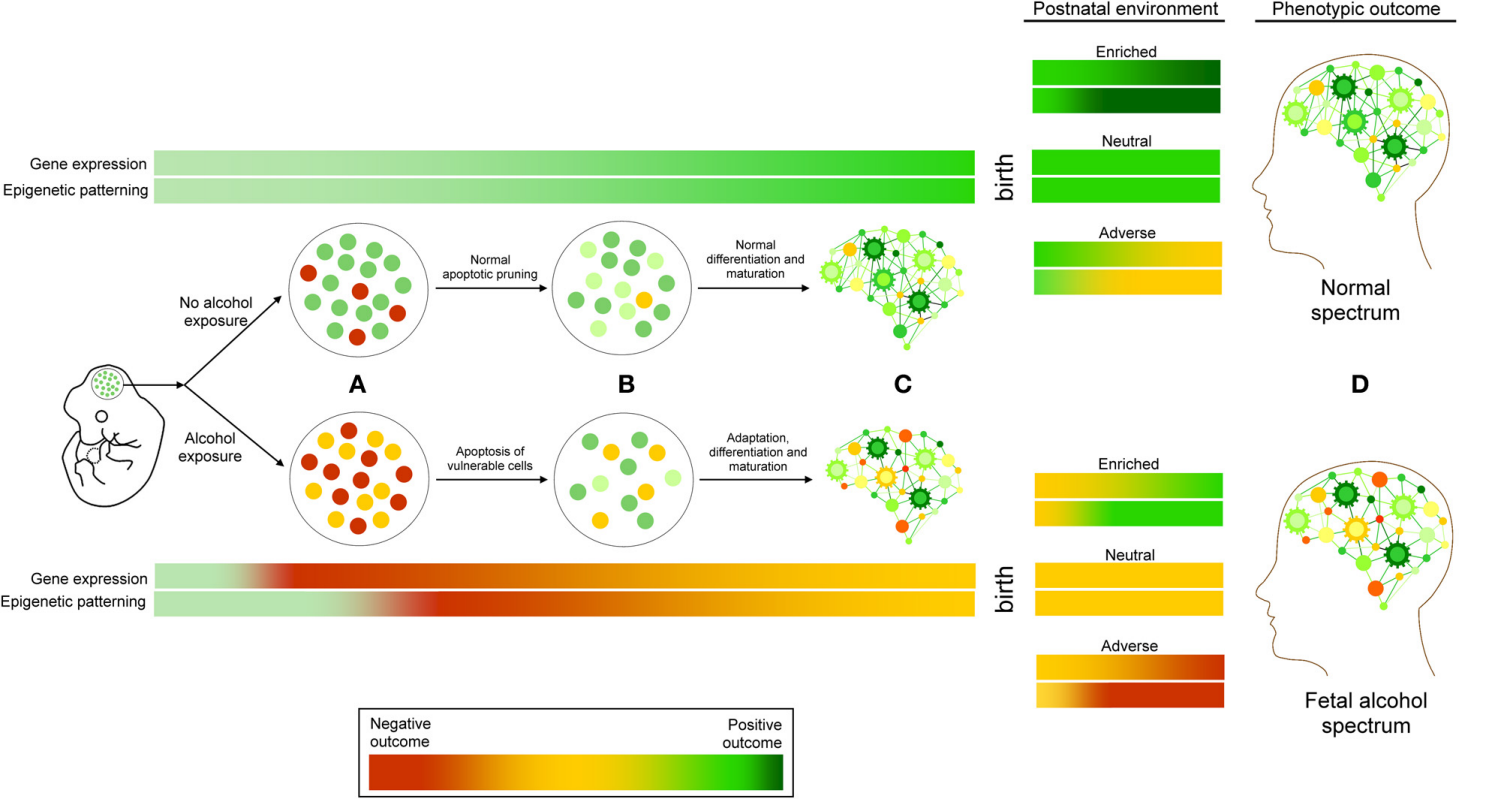
**A working model of FASD as a continuum of genetic and epigenetic events. (A)** Ethanol exposure results in cellular stress response, leading to the apoptosis of vulnerable cell types. **(B)** These changes are followed by molecular adaptations in surviving cells that include changes to epigenetic programming, including DNA methylation, histone modifications, and ncRNA regulation, which are **(C)** Subsequently inherited and maintained through cellular differentiation and maturation. These changes may be exacerbated or ameliorated by postnatal environmental conditions. Adverse (red) or positive (green) outcomes may depend on the interaction of these factors, contributing to the etiology of fetal alcohol spectrum disorders **(D)**.

Neurodevelopmental ethanol exposure results in a complex array of genetic and epigenetic changes in the brain. Currently, the results from studies examining these factors are varied, but consistent themes are emerging. They allow for the proposal of FASD as a continuum of molecular events (Figure 2). At the cellular level, it begins with neurotoxicity and ends with the selection and adaptation of those cells that comprise the adult neural population, resulting in life-long behavioral and cognitive changes. Ethanol exposure represents an interruption in normal neurodevelopmental processes. The surviving cells must adapt and acquire developmental trajectories that involve molecular adaptations that are detectable by genome-wide changes in gene expression and epigenetic patterning. These epigenetic alterations likely involve the interaction of DNA methylation, particularly in imprinted genomic regions, histone modification, and ncRNA regulation. These epigenetic changes are expected to be stably inherited following subsequent neurogenesis, differentiation, and maturation, and represent an enduring molecular “footprint” of neurodevelopmental ethanol exposure. This reprogramming of neurogenomic patterning may be further compounded by subsequent ontogenetic factors such as postnatal environment, further exacerbating or ameliorating epigenetic signatures. These signatures may—if shared by peripheral tissue sources—offer a source of early diagnosis of prenatal alcohol exposure. Also, if these changes may be mitigated by postnatal environmental interventions is an intriguing avenue for further investigation. Given the unlikelihood of total abstinence from alcohol consumption during pregnancy, evidence that the effects of prenatal alcohol exposure may be amended or reversed, both at the phenotypic and molecular level, would represent a significant step toward in improving the prognosis of individuals with FASD.

### Conflict of interest statement

The authors declare that the research was conducted in the absence of any commercial or financial relationships that could be construed as a potential conflict of interest.
